# Spud buds: stAST1 antagonizes potato tuber development by suppressing the alternative Tuberization Activation Complex (aTAC)

**DOI:** 10.1093/plphys/kiae180

**Published:** 2024-03-21

**Authors:** Kyle W Swentowsky

**Affiliations:** Assistant Features Editor, Plant Physiology, American Society of Plant Biologists; Cold Spring Harbor Laboratory, Cold Spring Harbor, NY, 11724, USA

The lifespan of an organism can be divided into a series of distinct developmental stages—for example, juvenility and adulthood. Life cycle transitions are therefore important for development, and of these, plant biologists are most familiar with the transition from vegetative to reproductive growth. The floral transition, as it is called, occurs in angiosperms to reach the reproductive stage and is regulated by florigen. The molecular identity of florigen was first identified in Arabidopsis as the FLOWERING LOCUS T (FT) protein, which has homologs in all other angiosperms that have been studied. Florigen is produced in the leaves in response to environmental and autonomous cues, then moves through the vascular system to cause the floral transition in the shoot apical meristem. Similarly, perennial plants must also use environmental cues to make decisions about when to produce storage organs that sequester the carbohydrates and nutrients necessary to survive dormant periods.

Potato (*Solanum tuberosum*) produces specialized subterranean storage organs called tubers. Because of its agronomic importance, potato has become a model system for tuberization, the process through which tuber developmental identity is established (reviewed in Zierer et al. 2021). Tuberization causes below-ground stolons to develop into tubers and is analogous to floral induction. SELF PRUNING 6A (StSP6A), a potato homolog of FT, is produced during short days and cool temperatures and moves to subapical stolons. There it interacts with a bZIP transcription factor to form the Tuberization Activation Complex (TAC), which activates the tuber gene expression program (Navarro et al. 2015). StSP6A also forms the alternative TAC (aTAC) through an interaction with a different bZIP protein that is involved in abscisic acid (ABA) signaling, StABI5-like 1 (StABL1) (Jing et al. 2022). The ratio of ABA and its antagonistic hormone, gibberellic acid (GA), is important for the stolon-to-tuber transition, but this process is not thoroughly understood at the molecular level.

Previously, Jing et al. (2022) performed ChIP-seq using StABL1 and discovered its binding sites contained many known binding motifs for another transcription factor family, the TCPs. This provided a clue that a TCP transcription factor may be involved in the regulation of aTAC gene targets. TCPs are well-known regulators of axillary branching, but some also affect floral transition. With the hint that a TCP member may be a part of the aTAC, Sun et al. (2024) first used a yeast two-hybrid assay with the known potato TCP proteins as prey to determine which members could interact with StSP6A and StABL1. One of these, StTCP23 interacted with both bait proteins and was expressed in developing tubers so was renamed to StSP6A-associated TCP protein 1 (StAST1).

A GUS-fusion line for *StAST1* was generated to analyze its gene expression pattern. *StAST1* is broadly expressed, particularly in the vasculature of most tissues. Importantly, *StAST1* is expressed in developing tubers and leaves, which overlaps the known expression pattern of *StSP6A*. Interestingly, *StAST1* had an opposite expression pattern compared to *StSP6A*, where its expression was higher at earlier tuberization stages and decreased during development when *StSP6A* expression peaks. Next, the authors tested the function of *StAST1* by increasing and decreasing its expression levels with 35S promoter (*OE-StAST1*) and RNAi (*Ri-StAST1*) transgenic lines, respectively, relative to the wild-type E3 line. In these experiments, plants with reduced *StAST1* expression produced more tubers, while elevated *StAST1* caused a reduction in tuber numbers, leading to the conclusion that *StAST1* negatively regulates tuberization (Figs. A and B). Interestingly, higher *StAST1* expression was correlated with a longer duration of the entire plant life cycle in these experiments, indicating that *StAST1* may affect tuberization by regulating plant maturity.

**Figure.**
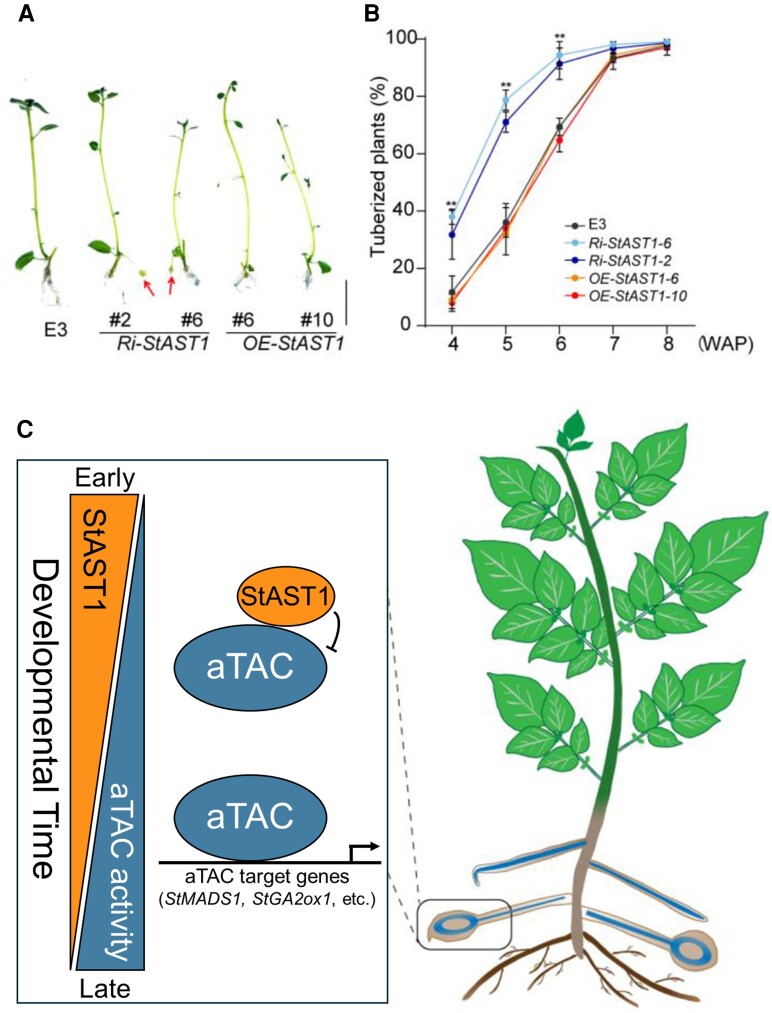
*StAST1* negatively regulates tuberization by suppressing the aTAC. **A)** Plants with decreased *StAST1* levels lowered by RNAi (*Ri-StAST1*) produce tubers sooner than the wild-type (E3) or plants overexpressing *StAST1* (*OE-StAst1*). **B)** Quantification of tuber development 4 to 8 weeks after pollination (WAP) in E3, *Ri-StAST1*, and *OE-StAST1* genotypes. **C)** A model to explain how StAST1 suppresses tuberization by attenuating the aTAC. StAST1 is expressed early in development, and it binds to the aTAC to suppress its activity StAST1 expression levels decrease during development, allowing the aTAC to promote expression of downstream genes. This complex binds to and regulates expression of many genes, including *StMADS1* and *StGA2ox1*, to direct tuber development in sub-apical stolons. Figures reproduced from Sun et al. 2024.

To understand the mechanism of how StAST1 affects tuberization, the authors next analyzed the protein interactions involved in forming the aTAC. Using yeast two-hybrid and BiFC experiments, it was found that only the C-terminal end of StAST1 is required to interact with the other members of the complex. A competitive dual-luficerase assay was also used to show that StAST1 could suppress the transcriptional activity of the aTAC, which is consistent with the role of *StAST1* as a negative regulator of tuberization by attenuating the aTAC.

Finally, a global view of how *StAST1* affects gene expression was gathered by performing RNA-seq in stolons of *StAST1-RNAi* plants. The majority of differentially expressed genes (1345/1906 genes) were downregulated. Among the most numerous classes of differentially expressed genes were those related to ABA signaling, and indeed it was found that *StAST1-RNAi* plants had significantly higher ABA levels in leaves and stolons; these plants were more sensitive to ABA in promoting tuber formation compared to wild-type controls. It was also observed that these had increased expression of genes involved in GA biosynthesis, which was consistent with higher levels of biologically active GA compounds in *StAST1-RNAi* plants. In processes where these two hormones are involved, ABA and GA often display an antagonistic relationship and it appears this antagonism likely contributes to tuber development. Expression of genes that control the mobilization of lipids and sugars, important metabolites stored within tubers, was also affected by *StAST1* expression.

The intricate regulatory network governing potato tuberization continues to unfold, revealing the interplay between environmental cues, hormone signaling, and transcriptional regulation. The discovery of *StAST1* as a negative regulator of tuberization sheds light on the fine-tuning mechanisms underlying this critical developmental process. Sun et al. (2024) speculate that StAST1 suppression of the aTAC evolved to fine-tune the timing of tuber development and present a model to explain the current understanding of this protein complex (Fig. C). When StAST1 is expressed early in development, it suppresses tuberization by binding StSP6A to inactivate the aTAC. StAST1 levels decrease during development, StSP6A forms the aTAC with StABL and 14-3-3 proteins, which activates *StMADS1* and *StGA2ox1* expression to drive tuber development.

As one of the world's most important food crops, potato plays a crucial role in addressing global hunger and nutrition. Understanding the molecular mechanisms underlying tuberization, a defining aspect of potato development, can lead to improved crop yields, enhanced resilience to environmental stresses, and increased agricultural productivity worldwide.
